# Attendance and Academic Performance in Neurophysiology: Engaging Dental Students Through Active Learning

**DOI:** 10.7759/cureus.115583

**Published:** 2026-09-01

**Authors:** Gustavo H Gameiro

**Affiliations:** 1 Department of Physiology, Universidade Federal do Rio Grande do Sul, Porto Alegre, BRA

**Keywords:** active learning, dental education, educational measurement, neurophysiology, school attendance

## Abstract

Engaging students in foundational neuroscience courses such as neurophysiology remains a challenge in dental education. Class attendance is often assumed to be associated with academic performance, yet evidence in basic science disciplines is inconsistent. This study analyzed the correlation between class attendance and academic performance among undergraduate dental students enrolled in a neurophysiology course. Institutional academic data from 185 students across four consecutive semesters (2023-2024) were retrospectively analyzed. The course included clinically oriented learning activities, such as case-based discussions involving interpretation of neuroanatomical magnetic resonance imaging (MRI) images, as well as a formative quiz administered in the week preceding the final examination. Attendance rates and final exam scores were correlated using Spearman’s test. Statistically significant positive correlations were observed in all semesters, with coefficients ranging from 0.37 to 0.56. These findings indicate that higher attendance was consistently associated with higher academic performance in a cognitively demanding foundational course. The results contribute to discussions on student engagement in dental education, with potential implications for student support and curricular planning in dental programs.

## Introduction

Student engagement and class attendance have long been considered relevant factors influencing academic performance in higher education, particularly in demanding foundational disciplines such as neurophysiology. In dental education, where basic sciences underpin the development of clinical competence, understanding the relationship between attendance and academic outcomes remains an important issue for curriculum planning. However, previous studies have reported inconsistent associations between lecture attendance and academic performance, suggesting that this relationship may depend on contextual and instructional factors rather than attendance alone [1-4].

Neurophysiology poses specific challenges to dental students due to its abstract concepts, complex neural pathways, and the need to integrate theoretical knowledge with clinical reasoning. The course accordingly incorporated active learning strategies, such as case-based learning and clinically contextualized activities, consistent with their increasing use in medical and dental education [5-13]. Although not directly evaluated in the present study, these strategies formed part of the instructional context in which class attendance was examined in relation to academic performance.

Despite the growing interest in active methodologies, few studies have specifically examined the association between class attendance and academic performance in basic science courses within undergraduate dental education. Existing evidence linking attendance to performance in dental education has largely focused on clinical or preclinical courses [3,4], or has been derived from broader reviews of teaching methods across health professions [1,2], with limited data available for foundational neuroscience-based disciplines such as neurophysiology, which combine abstract theoretical content with an increasing emphasis on clinical application. This gap limits understanding of whether attendance-performance associations described in other domains also apply to cognitively demanding basic science courses, and was the specific rationale for the present study.

Therefore, the present study aimed to analyze whether class attendance is associated with final examination performance among undergraduate dental students enrolled in a neurophysiology course, using data from four consecutive semesters.

## Materials and methods

Study design

This retrospective, observational study analyzed the correlation between class attendance and academic performance in a neurophysiology course offered to undergraduate dental students at the Federal University of Rio Grande do Sul (UFRGS), Porto Alegre, Brazil. The analysis included four consecutive cohorts across four academic semesters (2023-2024), totaling 185 students. The study population comprised students enrolled in the neurophysiology course during these four consecutive semesters (2023/1, 2023/2, 2024/1, and 2024/2). Students who withdrew from the course before completing the semester were not included in the analysis. These withdrawals were rare, with no more than one student excluded in any individual semester. The study was based exclusively on secondary institutional academic records, specifically attendance percentages and final examination grades. All data were fully anonymized prior to analysis, with no access to personal identifiers at any stage of the study. The data were originally collected as part of routine educational and administrative activities, without intervention, interaction, or prospective data collection involving students. In accordance with institutional guidelines and ethical standards for minimal-risk educational research using anonymized data, formal approval by a research ethics committee was not required. This is consistent with Brazilian National Health Council (CNS) Resolution No. 510/2016 (Article 1, sole paragraph, item II), which exempts research based exclusively on secondary, aggregated data with no possibility of individual identification from submission to the Research Ethics Committee (CEP)/National Research Ethics Commission (CONEP) ethics review system.

Course context and instructional structure

The neurophysiology module is part of the Physiology I curriculum and is typically delivered in the second semester of the dental program. Course content includes sensory and motor pathways, autonomic regulation, and oral physiology, with emphasis on mechanosensation, thermosensation, proprioception, and orofacial pain. Each instructional week consisted of a 90-minute expository lecture delivered in the morning to the full cohort (approximately 40 students), followed by 90-minute practical sessions conducted in the afternoon. To facilitate active participation, students were divided into two subgroups of approximately 20 students, scheduled at different times.

Practical sessions throughout the semester were primarily dedicated to case-based learning activities, conducted in small groups (five groups of four students). The same clinical cases were used across all groups and cohorts, focusing on the application of neurophysiological concepts to clinical reasoning, including the interpretation of neuroanatomical diagrams and corresponding magnetic resonance imaging (MRI) slices. No mandatory preparatory or extracurricular activities were required outside class time; self-directed study was encouraged but not formally structured or assessed.

Assessment and data analysis

A formative quiz was administered only in the week preceding the final examination, with the purpose of encouraging active recall and providing feedback prior to the summative assessment. Academic performance was measured by a single cumulative theoretical examination administered at the end of the semester. Although the specific questions varied across semesters, the examination followed the same blueprint, number of questions, topic weighting, and difficulty level in all four cohorts, supporting comparability of the outcome measure across semesters. Attendance was obtained from official course records, with attendance recorded manually by the instructor during each scheduled activity, and expressed as the percentage of sessions attended. The attendance percentage was calculated jointly from the weekly expository lectures and the corresponding practical (case-based) sessions relative to the total number of sessions offered during the semester. Data distribution for attendance and final grades was assessed using the Shapiro-Wilk test within each semester. As the Shapiro-Wilk test indicated a non-normal distribution for at least one of the two variables in every semester, Spearman’s rank-order correlation coefficient was used to examine the association between attendance and final grade in all four cohorts. Each semester was analyzed as an independent cohort; because the four correlations corresponded to distinct, non-overlapping groups of students rather than repeated tests of a single hypothesis, no correction for multiple comparisons (e.g., Bonferroni) was applied. Statistical analyses were performed using IBM SPSS Statistics for Windows, Version 27 (Released 2019; IBM Corp., Armonk, New York, United States), with significance set at p < 0.05.

## Results

The correlation between class attendance and final academic performance was analyzed for four consecutive cohorts of undergraduate dental students enrolled in the neurophysiology course from 2023 to 2024 (n = 185). Statistically significant positive correlations were observed in all four semesters, with correlation coefficients ranging from 0.37 to 0.56.

In the first semester analyzed (2023/1), the Spearman correlation coefficient was ρ = 0.56 (95% CI: 0.309-0.738), indicating a moderate positive correlation (p = 0.00012; n = 42). This same correlation strength (ρ = 0.56 (95% CI: 0.312-0.736)) was observed in 2023/2 (p = 0.0001; n = 43), suggesting consistent findings across different cohorts within the same year (Figure 1).

**Figure 1:**
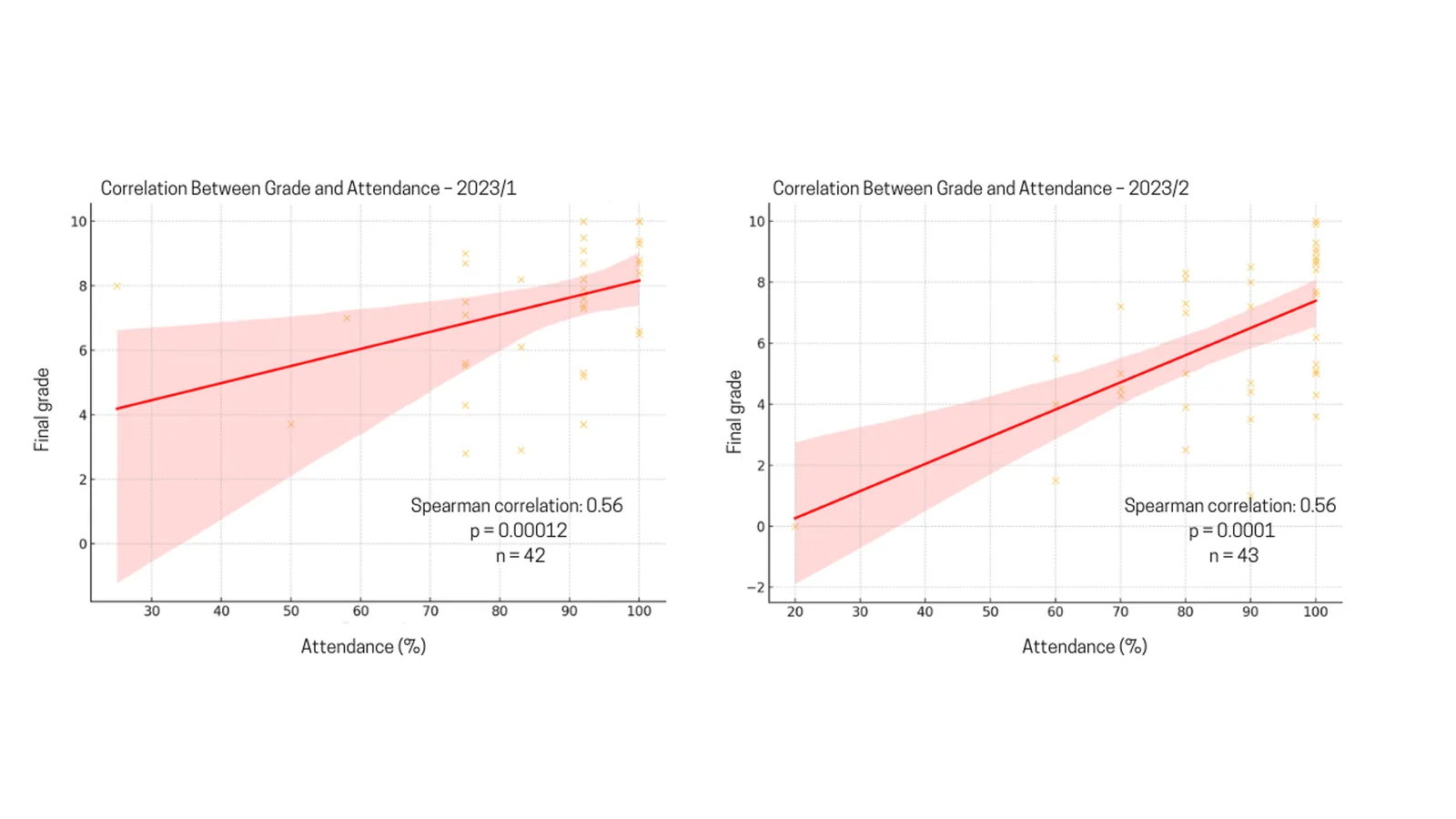
Scatterplots showing the correlation between class attendance (%) and final grade in the neurophysiology course across two consecutive semesters (year 2023). Semester 2023/1 (ρ = 0.56, p = 0.00012, n = 42) and Semester 2023/2 (ρ = 0.56, p = 0.0001, n = 43). Trend lines represent Spearman’s rank-order correlations, with shaded areas indicating 95% confidence intervals.

In 2024/1, a positive but slightly weaker correlation was identified (ρ = 0.37 (95% CI: 0.119-0.577), p = 0.005; n = 56), followed by a similar trend in 2024/2 with ρ = 0.41 (95% CI: 0.129-0.630) (p = 0.005; n = 44), both still reaching statistical significance (Figure 2).

**Figure 2:**
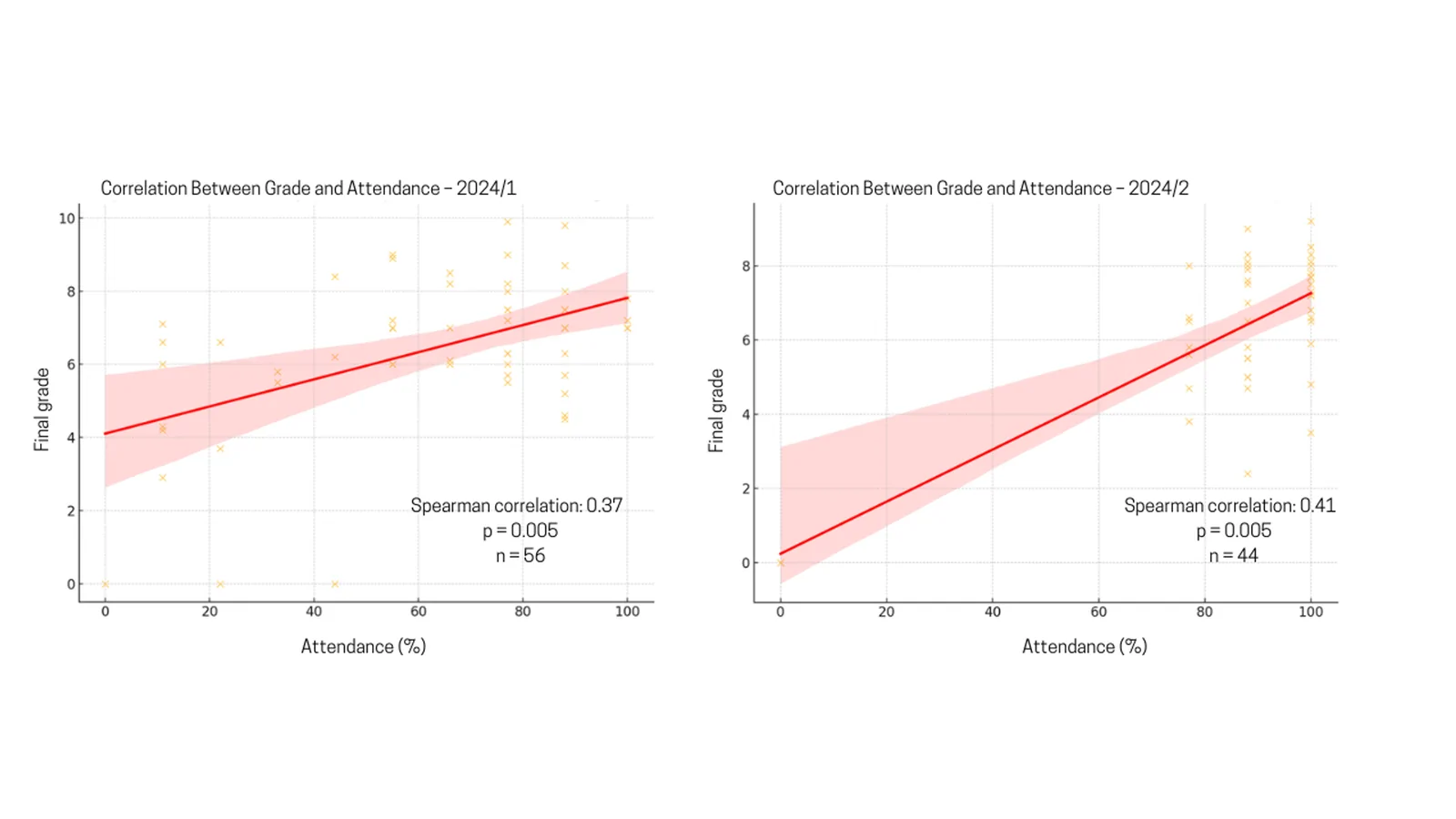
Scatterplots showing the correlation between class attendance (%) and final grade in the neurophysiology course across two consecutive semesters (year 2024). Semester 2024/1 (ρ = 0.37, p = 0.005, n = 56) and Semester 2024/2 (ρ = 0.41, p = 0.005, n = 44). Trend lines represent Spearman’s rank-order correlations, with shaded areas indicating 95% confidence intervals.

Across all semesters, higher attendance rates were consistently associated with higher final grades, indicating a consistent positive relationship between class participation and academic performance, even when only one cumulative exam was used for performance evaluation.

To contextualize these findings, the mean attendance and final grades for each cohort are presented in Table 1. It should be noted that the comparatively lower mean attendance observed in 2024/1 (64.8 ± 27.1%) coincided with the severe flooding that affected Rio Grande do Sul during that semester, which led to the temporary replacement of some in-person classes with synchronous online sessions. Although the individual impact of this disruption on attendance could not be quantified retrospectively, it represents a plausible contextual factor contributing to the lower attendance observed in this cohort.

**Table 1:** Mean and standard deviation (SD) of class attendance percentages and final exam scores for undergraduate dental students enrolled in the neurophysiology course over four consecutive semesters.

Semester	Attendance Percentages (Mean ± SD)	Final Grades (Mean ± SD)
2023/1	85.4 ± 17.2	6.68 ± 2.06
2023/2	89.5 ± 13.7	6.84 ± 2.23
2024/1	64.8 ± 27.1	6.14 ± 1.99
2024/2	85.9 ± 20.6	6.52 ± 2.00

## Discussion

The present study demonstrated a consistent and statistically significant positive correlation between class attendance and academic performance in a neurophysiology course for undergraduate dental students across four consecutive semesters. These findings indicate that higher attendance was associated with higher final exam scores, consistent with an association between sustained class participation and performance in cognitively demanding foundational disciplines.

It is important to emphasize that the present analysis does not evaluate the isolated or comparative effect of specific teaching strategies on academic performance. Rather, the observed association reflects the relationship between attendance and performance within a defined instructional context. The course incorporated clinically oriented activities, such as case-based learning and a formative quiz prior to the final examination, which were designed to promote engagement and support knowledge integration. Previous studies in medical and dental education suggest that such strategies may enhance motivation, active recall, and contextual learning, potentially contributing to student engagement and persistence in classroom activities [1,8,13]. However, in the absence of a control group or comparative instructional model, no causal inferences regarding the impact of these strategies on grades can be drawn from the present data.

From a theoretical perspective, engagement-centered learning environments may facilitate knowledge integration by encouraging meaningful connections between new information and existing cognitive structures, particularly in complex domains such as neurophysiology. Educational theories of meaningful learning and evidence from educational neuroscience emphasize the role of contextualization, emotional relevance, and retrieval practice in supporting durable learning [14-17]. While these frameworks help interpret the educational context in which the present findings were observed, they were not directly assessed in this study and should therefore be viewed as complementary explanatory models rather than empirical outcomes. Accordingly, these theoretical frameworks should not be interpreted as explaining the observed correlation, which reflects an association between attendance and performance rather than evidence attributable to any specific instructional strategy or learning mechanism.

This study has some limitations that should be acknowledged. Its retrospective design and reliance on secondary institutional data preclude the assessment of causal relationships. The analysis was conducted in a single institution and used a single summative examination as the outcome measure. In addition, individual levels of engagement during classroom activities were not directly measured, nor were alternative instructional models or student perceptions formally evaluated. Furthermore, the observed association between attendance and examination performance may be influenced by unmeasured factors such as prior academic ability, motivation, study habits, or student engagement, which were not captured in the institutional records used in this study. Accordingly, attendance should not be interpreted as an independent determinant of academic performance, but rather as one of several interrelated factors associated with it. Future studies comparing different teaching approaches, incorporating qualitative measures of student engagement, or using longitudinal and multicenter designs may help clarify the mechanisms underlying the relationship between attendance, engagement, and academic performance.

## Conclusions

This study demonstrated a consistent positive correlation between class attendance and academic performance among undergraduate dental students enrolled in a neurophysiology course across four consecutive semesters. The findings indicate that higher attendance was consistently associated with higher final examination scores in a cognitively demanding foundational discipline within dental education, although the underlying mechanisms linking attendance to performance remain to be clarified. Although conducted in a course that incorporated clinically oriented learning activities, the results do not support causal inferences regarding specific teaching strategies. Instead, the study contributes empirical evidence to discussions on student engagement in basic science courses and highlights attendance as a relevant factor associated with academic outcomes in undergraduate dental curricula.
